# Network Pharmacology and Intestinal Microbiota Analysis Revealing the Mechanism of Punicalagin Improving Bacterial Enteritis

**DOI:** 10.2174/1573409919666230526165501

**Published:** 2023-09-22

**Authors:** Shuyun Huang, Ying Wang, Qingsong Zhu, Hongmin Guo, Zongyuan Hong, Shuzhi Zhong

**Affiliations:** 1 Tissue and Embryo Department, Wannan Medical College, Wuhu, 241002, China;; 2 Computer and Information Department, Hohai University, Nanjing, 210024, China

**Keywords:** Punicalagin, bacterial enteritis, network pharmacology, intestinal flora, molecular docking, anti-inflammatory

## Abstract

**Background:**

The Chinese medicine *punicalagin* (Pun), the most important active ingredient in pomegranate peel, has significant bacteriostatic and anti-inflammatory properties. The potential mechanisms of Pun for bacterial enteritis, however, are unknown.

**Objective:**

The goal of our research is to investigate the mechanism of Pun in the treatment of bacterial enteritis using computer-aided drug technology, as well as to investigate the intervention effect of Pun on mice with bacterial enteritis using intestinal flora sequencing.

**Methods:**

The targets of Pun and Bacterial enteritis were obtained by using the specific database, and cross-targets were screened among these targets, followed by PPI and enrichment analysis of the targets. Furthermore, the degree of binding between Pun and key targets was predicted through molecular docking. After successfully establishing the bacterial enteritis model *in vivo*, mice were randomly assigned to groups. They were treated for 7 days, the symptoms were observed daily, and the daily DAI and body weight change rate were calculated. Following administration, the intestinal tissue was removed, and the contents were separated. The tight junction protein expression was detected in the small intestine by the immunohistochemical method; ELISA and Western Blot (WB) were performed to detect the expressions of tumor necrosis factor-α (TNF-α) and interleukin-6 (IL-6) in the serum and intestinal wall of mice. The 16S rRNA sequence was used to determine the composition and diversity of the intestinal flora of mice.

**Results:**

In total, 130 intersection targets of Pun and disease were screened by network pharmacology. The enrichment analysis showed cross genes were closely related and enriched in the cancer regulation and the TNF signal pathway. The active components of Pun could specifically bind to the core targets TNF, IL-6, *etc*., determined from molecular docking results. *In vivo* experiment results showed that the symptoms in the PUN group mice were alleviated, and the expression levels of TNF-α and IL-6 were significantly reduced. A Pun can cause substantial changes in the intestinal flora of mice in terms of structure and function.

**Conclusion:**

Pun plays a multi-target role in alleviating bacterial enteritis by regulating intestinal flora.

## 1 INTRODUCTION

Bacterial enteritis is one of the most common digestive tract infections in the clinic. The disease has a high incidence in children, and the main symptoms include diarrhea, fecal odor, and mucus [1, 2]. Antibiotics are mainly used in clinical treatment. Due to the physiological characteristics of the digestive tract of children, the administration of antibiotics for a long time makes them prone to antibiotic-related enteritis and also increases bacterial drug resistance. It is often necessary to replace sensitive antibiotics, which can further lead to intestinal microbiological imbalance and aggravate enteritis and diarrhea [3]. Therefore, a more effective treatment is required. Punicalagin (Pun) is the main active component extracted from the pomegranate peel [4]. Pun is a traditional Chinese medicine that is common and has many effects, such as antibacterial, anti-inflammatory, and antioxidant. According to the TCM theory, Puns can be used in the treatment of diseases characterized by epigastric cold, pain, and dyspepsia [5, 6]. Pun shows selective toxicity toward intestinal pathogenic bacteria and can improve the imbalance of intestinal bacteria caused by stress and other factors [7, 8]. In this study, we investigated the components of Pun, which are primarily active and used to treat bacterial enteritis, predicting their targets of action and the related signaling pathways. This was accomplished through the use of computer-aided drug technology, such as network pharmacology and molecular docking. The possible mechanism by which Pun treats bacterial enteritis was then determined, and the effects of Pun on the intestinal flora were studied using intestinal flora sequencing. These results provided information regarding the mechanism of Pun that might help further develop and utilize Pun in treating bacterial enteritis.

## 2 MATERIALS AND METHODS

### 2.1 Network Pharmacology Section

#### 2.1.1 Pun and Bacterial Enteritis Common Target

The six active ingredients, which were in Pun, were obtained by using the Traditional Chinese Medicine Systems Pharmacology Database and Analysis Platform database (TCMSP) (Table **1**) [9]. The targets of the drugs were screened using “Oral Bioavailability (OB) ≥30% and Drug Likeness (DL) ≥0.18” as criteria [10]. Using the UniProt database, we standardized the names of the selected targets and created a dataset of the drug's active ingredient targets. The GeneCards database was searched with “bacterial enteritis” as the keyword to obtain the relevant targets of bacterial enteritis, and a Venny analysis was performed with the corresponding targets of Pun. The intersection of the target of Pun and bacterial enteritis in the diagram was the potential target, and the anti-disease target dataset of the active components of the drugs was generated.

#### 2.1.2 Constructing the PPI Network and Analyzing the Core Target

To elucidate interactions between cross-targets, the Drug and disease cross-target data were imported into the STRING database, “Homo sapiens” was used as the species, “highest confidence (>0.4)” was used as the minimum interaction threshold, and the remaining parameters remained at their original default settings [11]. Then, saved the protein-protein interaction (PPI) network of the obtained intersecting target in “TSV” format. The Cytoscape 3.9.0 software was used for topological analysis to determine the genes whose geneticity value exceeded the average score. A bar chart was generated in R3.6.0 and exported in the PNG file format. The core PPI network was further analyzed through the “MCODE plug-in”, which was in the Cytoscape 3.9.0 software.

#### 2.1.3 Functional Enrichment Analysis

The results of the Gene Ontology (GO) function analysis [12] and the Kyoto Encyclopedia of Genes and Genomes (KEGG) pathway analysis [13] of the intersection targets were obtained by analyzing the data in the DAVID database. The intersection target was imported into the gene list of the DAVID database, the “OFFICIAL-GENE-SYMBOL” was selected, “Homo sapiens” was used as the species, exported the data, and the GO function and the KEGG pathway analyses were performed. The top biological process (BP), Cellular Component (CC), Molecular function (MF), and pathway were screened, and the data were visualized.

### 2.2 Molecular Docking Section

At first, the 2D structure of Pun was obtained from the PubChem database, and the 3D structure of the active ingredient was downloaded from the RCSB PDB database. The software AutoDock Vina was then used to create the ligands and proteins needed for molecular docking [14]. Pretreatment was performed to analyze the crystal structure of target proteins, including hydrogenation, modification of amino acids, optimization of energy, removal of water molecules, and adjustment of force field parameters. Subsequently, the low-energy conformation of the ligand structure was met. Finally, the five key target structures were docked with the structure of one active component and the 3D diagram was constructed using the PyMOL 2.2.0 software, and the 2D diagram was constructed using Maestro elements.

### 2.3 Animal Experiment Section

#### 2.3.1 Chemicals and Reagents

*Punicalagin* powder (PUN, purity HPLC ≥ 98%) was supplied by Yuanye Biotechnology (Shanghai, China). Cefixime granules (CEF) were purchased from Baiyunshan Pharmaceuticals (Guangzhou, Guangdong, China). Pathogenic *Escherichia coli* O_101_ was purchased from Beina Biotechnology (Henan, China). The tumor necrosis factor-α (TNF-α) and interleukin-6 (IL-6) ELISA kits were purchased from Lianke Biotechnology (Hangzhou, Zhejiang, China). TNF-α, IL-6, β-Actin, occludin, and ZO-1 anti-rabbit antibodies were supplied by Abcam Technology (UK). Beyotime Biotechnology provided the horseradish peroxidase secondary antibody, bicinchoninic acid protein assay kit (BCA), RIPA lysis buffer, protein-loading buffer, and Hematoxylin and Eosin staining kit (HE Staining Kit) (Shanghai, China).

#### 2.3.2 Animal Sample Collection

Sixty BALB/c mice (22 ± 3 g) with the animal license SCXK (Su) 2021-0001 were obtained from the Qinglongshan animal breeding base (Nanjing, Jiangsu, China). The mice were eight weeks old and were split evenly between male and female mice. All mice were SPF grade and raised in the animal center of Wannan South Medical College. The environment of this condition is as follows: humidity is 40-70%, and room temperature is 23 ± 2°C. Changing lighting conditions, unrestricted consumption of food and liquids. The experiment was initiated one week after adaptive feeding.

The cultures of pathogenic *Escherichia coli* O_101_ liquid were incubated at 180 r/min and 37°C in a shaking incubator for 24 h. Centrifuged the bacterial solution to collect the bacteria at 5000 rpm for 5 min and washed three times with PBS. The bacterial pellet was then resuspended in PBS, eventually adjusting the concentration to 2.6×10^8^ CFU/mL. Forty-five BALB/c mice were taken, and the prepared pathogenic *E. coli* O_101_ suspension was gavaged in 10 mL·kg^–1^, which was to achieve the enteritis disease model [15]. The enteritis model mice, at random, were divided into the following groups: Model group, PUN group, and CEF group. The administration group was gavaged with Pun (25mg·kg^–1^) and CEF (3mg·kg^-1^) from the day of modeling for seven consecutive days. Another group of normal mice (n = 15) was used as the control group. Each group of mice consisted of half males and half females. The treatment lasted 7 days, and the body weights of all mice were recorded every day at 10 a.m. The Wannan Medical College Ethics Committee approved the animal protocols in this study under the approval number LLSC-2021-141, and they also meet the requirements of the National Institutes of Health's Guidelines for the Care and Use of Laboratory Animals.

#### 2.3.3 Immunohistochemistry

Using hematoxylin and eosin, the small intestines were stained and embedded in paraffin and cut into 5 µm thick segments. Another set of intestinal tissue sections (5 µm thick) was cut, H_2_O_2_ was added at room temperature for 10 min, and rinsed. Then, 5% BSA was added and incubated for 60 min at room temperature. The rabbit anti-mouse primary antibody occludin and ZO-1 were added and incubated overnight at 4°C. The next day, the slices were rinsed under clean water, and added the secondary antibody, biotinylated sheep anti-rabbit IgG at room temperature, and incubated for 60 min. After rinsing the sections, the streptavidin-biotin peroxidase complex solution (SABC 1:100) was added, and DAB was used to develop color at room temperature for 60 min.

#### 2.3.4 ELISA

Blood was drawn from each group of mice, and serum samples were isolated. The TNF-α and IL-6 levels were then determined using the ELISA kit.

#### 2.3.5 16S rRNA Sequencing

The total RNA of bacteria was extracted from the feces of mice in each group. RNA samples (10 ng) were taken, and PCR amplification was performed with specific primers with a barcode according to the sequence of the specific region of the 16S rRNA [16]. The library was constructed using the library building kit, quantified by Qubit and qPCR, and sequenced on the computer after the library was qualified. The data were obtained, the representative sequence of OTUs was selected, and the characteristic abundance was normalized. The technology was provided by the Hangzhou Lianchuan Biotechnology Company.

#### 2.3.6 Western Blot Assay

The tissue was placed in an EP tube and lysed on ice by adding the lysis buffer (containing 1% PMSF, a protease inhibitor) for 40 min. The lysate was collected, mixed with 2x load buffer (Beyotime), and separated by SDS-PAGE (20 µL/well). The protein was transferred onto a PVDF membrane, blocked by using a rapid blocking solution for 20 min, incubated at 4°C conditions in primary antibodies overnight, the next day, and then incubated with secondary antibodies for 1h. The results were observed using the Odyssey imaging system (LI-COR).

#### 2.3.7 Statistical Processing

All data were reported as the mean ± standard deviation. T-tests and one-way analysis of variance were used to compare the two groups (ANOVA). At *p* <0.05, all differences within and between groups were considered statistically significant. The data were statistically analyzed using SPSS18.0.

## 3 RESULTS

### 3.1 Drug Target Disease Interaction Network

**Fig. (1):**
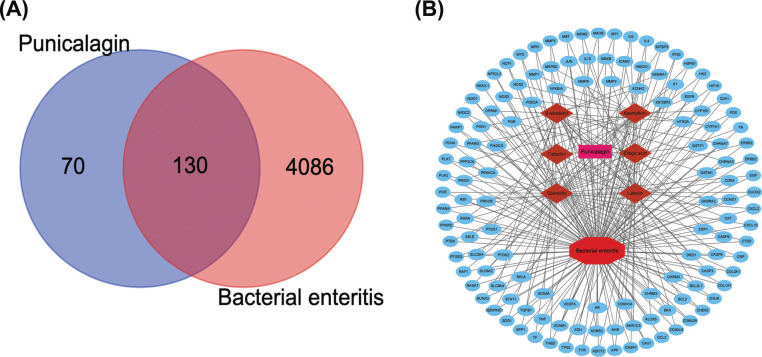
The interaction network of drug targets and disease targets. (**A**) The venn diagram of drug and disease targets. (**B**) The visualization of the network of targets of Pun after the treatment of bacterial enteritis; The red polygons represent diseases, the pink rectangle represents drugs, the blue ellipses are gene targets, the orange diamond is the active ingredient of the medicine, and the lines represent interactions.

The six active ingredients of Pun were obtained from the TCMSP database (Table **2**), 200 corresponding targets of Pun were obtained by searching for standardized protein targets in the UniProt protein database, and 4,216 bacterial enteritis targets were obtained from the database which was GeneCards. Using the online software Venny 2.1.0, the corresponding targets of the disease and the drug were intersected, and 130 antibacterial enteritis targets of Pun were obtained (Fig. **1A**). The network of common targets was then visualized, allowing drugs, diseases, and targets to be displayed more intuitively (Fig. **1B**).

### 3.2 Constructing the PPI Network and Analyzing the Key Target

**Fig. (2):**
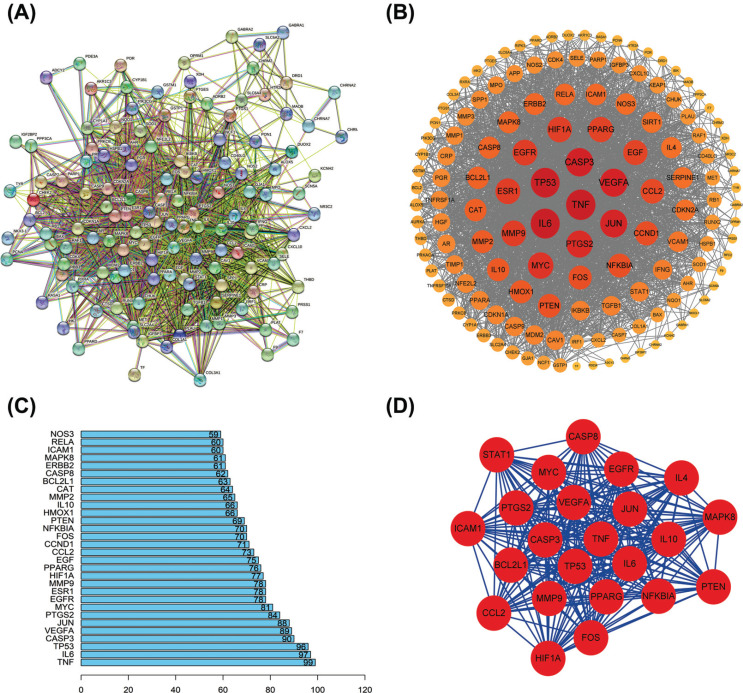
PPI enrichment analysis. (**A** and **B**): The protein interaction network diagram; Node size and color depth represent degree values, and the size of the node and the shade of the color are proportional to the degree value. (**C**) PPI cluster analysis. (**D**) The core network was constructed based on the PPI topology analysis.

A total of 130 targets were entered into the STRING database, and the PPI network was drawn using Cytoscape software. According to the network, the cross genes for Pun and bacterial enteritis were highly correlated (Fig. **2A**). Then, to visualize relationships, we created networks of common targets (Fig. **2B**). The software was also used to conduct topology analysis, which was critical for the PPI network. The first 30 targets were plotted in R 3.6.1 (Fig. **2C**). TNF-α (TNF-α), interleukin-6 (IL6), tumor suppressor p53 (TP53), caspase-3 (CASP-3), and vascular endothelial growth factor A were the core targets with the highest degree value (VEGFA). These five targets might be the key targets associated with the treatment of bacterial enteritis using PUN. The PPI network was then used for gene cluster analysis and core target screening with the MCODE module, which was included in the Cytoscape 3.7.2 software (Fig. **2D**).

### 3.3 Functional Enrichment Analysis

**Fig. (3):**
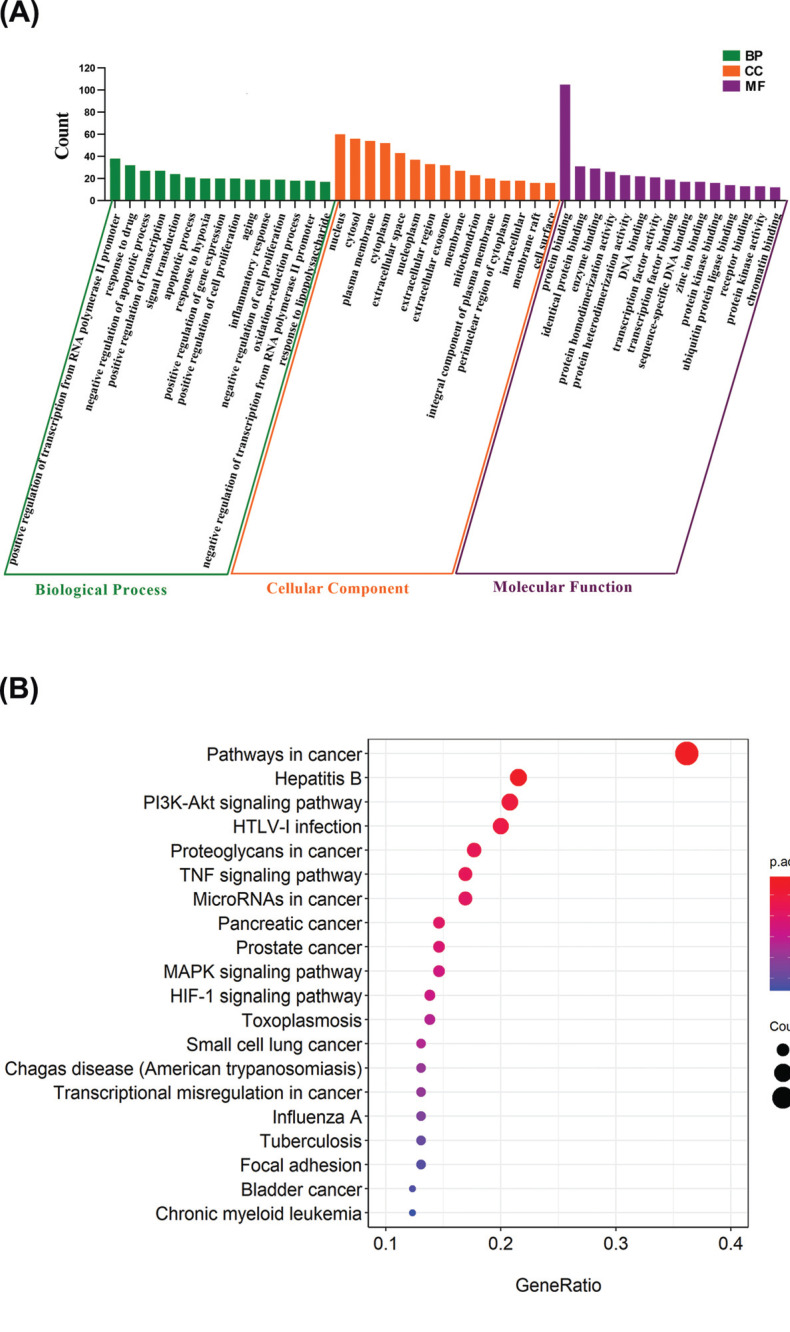
The results of the functional enrichment analysis. (**A**) The GO analysis and (**B**) the KEGG pathway.

DAVID database was used for GO enrichment analysis of the intersection targets, which were PUN targets for treating bacterial enteritis. Through systematic analysis, we found that in the biological process of the treatment of bacterial enteritis with Pun, there was a positive regulation of transcription from the RNA polymerase II promoter, response to the drug, negative regulation of the apoptotic process, positive regulation of transcription, DNA-templated, signal transduction, and the apoptotic process. Cellular components consist of the nucleus, cytosol, plasma membrane, cytoplasm, extracellular space, *etc*. Among their molecular functions, protein binding, identity protein binding, enzyme binding, and protein homodimerization activity were the primary functions (Fig. **3A**). The KEGG signaling pathway analysis of the core target of PUN for treating bacterial enteritis was performed by analyzing the data in the DAVID database. All signal pathways were screened, and the results were visualized using the WeChat messaging platform and the research status of bacterial enteritis (Fig. **3B**). The results showed that the enriched signal pathways were mainly related to bacterial enteritis, including pathways in cancer, the PI3K-Akt and the TNF signaling pathway, MicroRNAs in cancer, pancreatic cancer, prostate cancer, and the MAPK signaling pathway. The results showed the multi-channel and multi-target role of Pun in the treatment of bacterial enteritis.

### 3.4 Molecular Docking

**Fig. (5):**
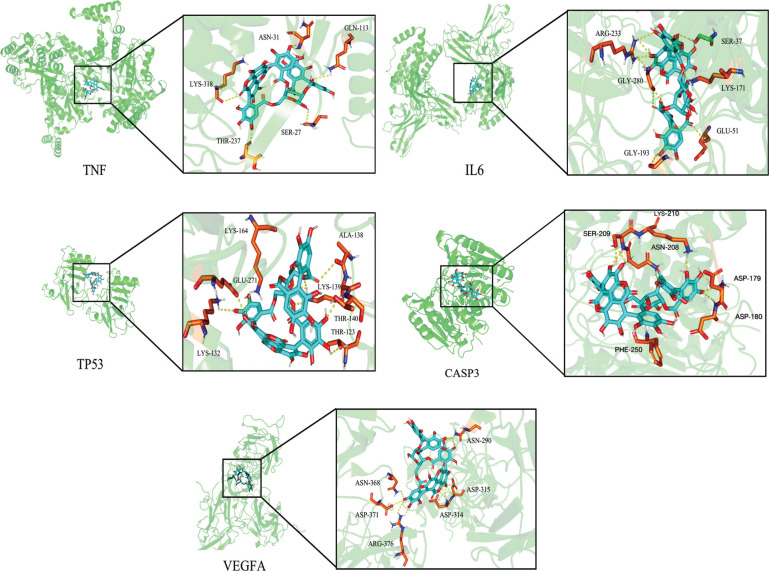
The docking mode diagram of Pun and the core target molecules.

The results of network pharmacology were further validated; the targets with a higher degree, including TNF, IL6, TP53, CASP3, and VEGFA, were selected for molecular docking among the common targets of drugs and diseases The molecular structure of Pun was shown in Figs. (**4A** and **B**). Higher conformational stability is associated with lower energy and a greater possibility of interaction when the ligand and receptor are combined. The energy (kcal/mol) value represents the binding ability of the two. Lower binding ability is associated with more stable binding between the ligand and the receptor, as well as higher molecular conformation stability. Generally, binding energy <0 indicates that ligand molecules can freely bind to the receptor protein. Its absolute value >5 represents better binding activity, and an absolute value >7 represents strong binding activity [17]. The molecular docking results showed a strong binding force between Pun and TNF, IL6, TP53, CASP3, and VEGFA. Among these, TNF had the highest binding force (Table **3**). Pun formed hydrogen bonds with ASN31, GLN113, LYS318, THR237, and SER27 of TNF; with SER37, LYS171, GLU51, GLY193, GLY280, and ARG233 of IL-6; with LYS164, ALA138, LYS139, THR140, THR123, LYS132, and GLU271 of TP53; with LYS210, ASN208, ASP179, ASP180, PHE250, and SER209 of CASP3; with ASN290, ASP315, ASP314, ARG376, ASP371, and ASN368 of VEGFA (Fig. **5**).

### 3.5 Animal Experiment

#### 3.5.1 Effect of Escherichia coli on Mice

**Fig. (6):**
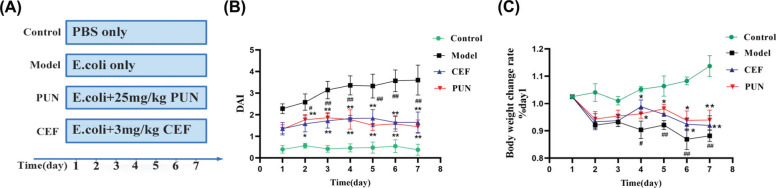
The establishment of the mouse model of bacterial enteritis. (**A**) Experimental design, (**B**) Weight change rate, and (**C**) The results of DAI. Statistical significance: ^#^*p* < 0.05 and ^##^*p* < 0.01 compared with control group; *p < 0.05 and **p < 0.01 compared with model group (n = 6).

Diarrhea symptoms appeared about 12 h after bacterial infection, including mental fatigue, loss of appetite, dull hair, yellow loose stool, and secretion outflow (Fig. **6A**). The model group mice had a lesser appetite, decreased activity, dull hair color, and lower weight than those in the control group. On the fourth day of administration, the weight loss in the control group was statistically significant (*p* <0.05). When compared to the model group, the mice in the PUN and CEF groups gained significantly more weight on the fifth day of administration (*p* <0.05) (Fig. **6B**). From the start of modeling, the mice in each group were scored by disease activity index (DAI) at a set time every day. The DAI index was calculated by taking measurements of the weight, stool characteristics, and blood in the stool of mice [18]. The model group's score increased significantly from the second day when compared to the control group (*p* <0.05) (Fig. **6C**). When compared to the model mice, the PUN and CEF groups had significantly lower scores (*p* <0.05).

#### 3.5.2 Effect of Pun on the Intestinal Epithelium of Mice with Enteritis

**Fig. (7):**
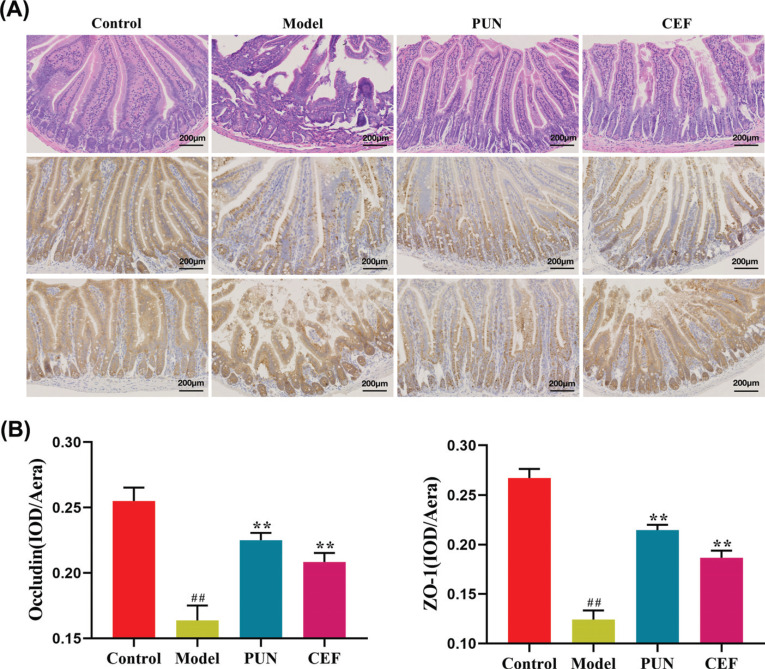
The effect of Pun on the intestinal epithelium of mice with enteritis. (**A**) The H&E staining and immunohistochemical map of occludin and ZO-1. (**B**) Bar charts. Statistical significance: ^##^*p* < 0.01 compared with the control group; **p < 0.01 compared with the model group (n = 6).

The H&E stained sections showed that the intestinal mucosa of the control group was intact without ulcers, the villi of the small intestine were arranged closely and regularly, and the morphology of the epithelial cells was normal (Fig. **7A**). The intestinal wall of the model group had conspicuous ulcers, lower villi density, more disordered villi, and more shedding of the epithelial cells compared to the condition of the intestinal wall of the control group. The intestinal glands and the epithelium of the mucosal layer were completely lost, accompanied by the infiltration of many inflammatory cells, and the inflammation invaded the submucosa and muscle layer. The epithelial cells in the intestinal wall of the mice in the PUN and CEF groups were shed to a lesser extent, the villi were arranged regularly, the infiltration of the inflammatory cells was lower, and the symptoms of the infection were significantly reduced. The tight junction protein occludin and ZO-1 are important components in forming tight junctions between intestinal epithelial cells. It is mainly expressed in the cytoplasm of the lateral top epithelial cells, maintaining the integrity of the intestinal wall. The images obtained by histochemical staining showed brown regions that indicated the positive expression of occludin and ZO-1. The expression of occludin proteins in the intestinal wall of mice in the model group was significantly lower than in the control group (*p* <0.05), and the occludin and ZO-1 proteins in the medication group were weakly expressed compared to the model group (*p* <0.05) (Fig. **7B**).

#### 3.5.3 Effect of Pun on the Core Targets TNF-α and IL6 in Enteritis Mice

**Fig. (8):**
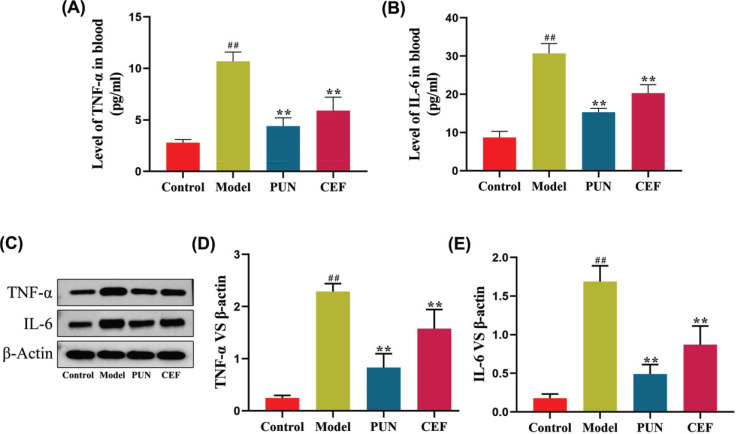
The effect of Pun on the core targets TNF-α and IL6 in enteritis mice. (**A** and **B**) The results of ELISA. (**C** and **D**) The results of the WB assay. Statistical significance: ^##^*p* < 0.01 compared with the control group; **p < 0.01 compared with the model group (n = 6).

The TNF-α and IL-6 levels in the serum of mice in the model group were significantly higher than those in the control group (*p* < 0.01) (Figs. **8A** and **B**). TNF-α and IL-6 levels in the treatment group mice were significantly lower than those in the model group (*p* < 0.01). The TNF-α and IL-6 levels in the intestinal wall of the mice in the model group were significantly higher than those in the control group (*p* < 0.01) (Figs. **8C** and **D**). TNF-α and IL-6 levels in the treatment group mice were significantly lower than their levels in the model group mice (*p* < 0.01).

### 3.6 Sequencing the Intestinal Flora to Elucidate the Mechanism of Pun in the Treatment of Enteritis

**Fig. (9):**
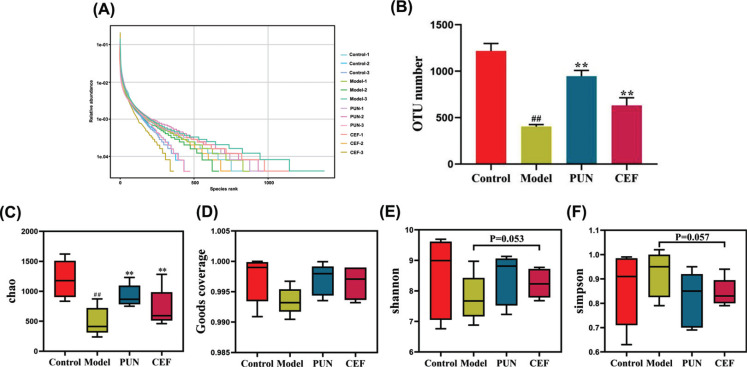
Changes in the intestinal microbial diversity after PUN treatment. (**A**) Species-rank curve, (**B**) OTU number. (**C**-**F**) The α-diversity indices included (**C**) Chao, (**D**) Goods coverage, (**E**) Shannon, and (**F**) Simpson indices. Statistical significance: ^##^*p* < 0.01 compared with the control group; **p < 0.01 compared with the model group (n = 6).

In terms of species richness and evenness, the indices of the model group were lower than those of the other 3 groups, as indicated by the OTU curve (Fig. **9A**). Specifically, the number of OTUs (Fig. **9B**), observed Chao (Fig. **9C**), and good coverage (Fig. **9D**) in the model group were significantly lower than those in the control group (*p* < 0.05). After PUN treatment, the decline in the α diversity index returned to the control group level (*p* < 0.05). There were no differences in the Shannon and Simpson values among the groups (Fig. **9E**, *p* = 0.053; Fig. **9F**, *p* = 0.057). The convergence of the above four indicators indicated that the experimental data included all species in the community.

**Fig. (10):**
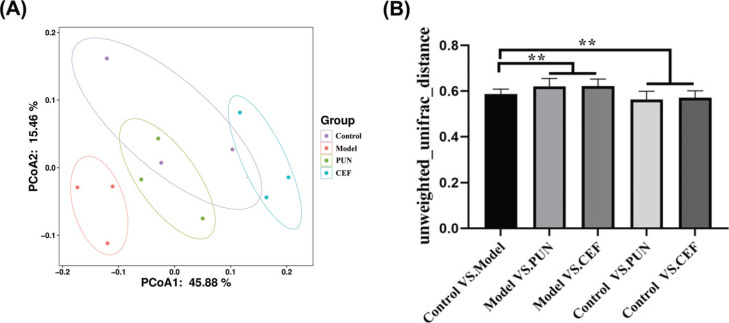
The changes in the β-diversity of intestinal microbiota after PUN treatment. (**A**) PCOA and (**B**) the unweighted uniFrac distance. Statistical significance: **p < 0.01 compared with the model group (n = 6).

Subsequently, the PUN and Control groups were evaluated by the PCoA method based on the differences in β-diversity. The PCoA diagram showed that the intestinal microbiota in the model group changed significantly, and the intestinal microbiota in the model group was obviously distant from the other three groups (Fig. **10A**). Fig. (**10B**) shows the differences between the Control, PUN, and CEF groups. The results indicated that the intestinal flora of the model group and the PUN treatment group has an obvious difference from that of the control group (*p* < 0.05).

**Fig. (11):**
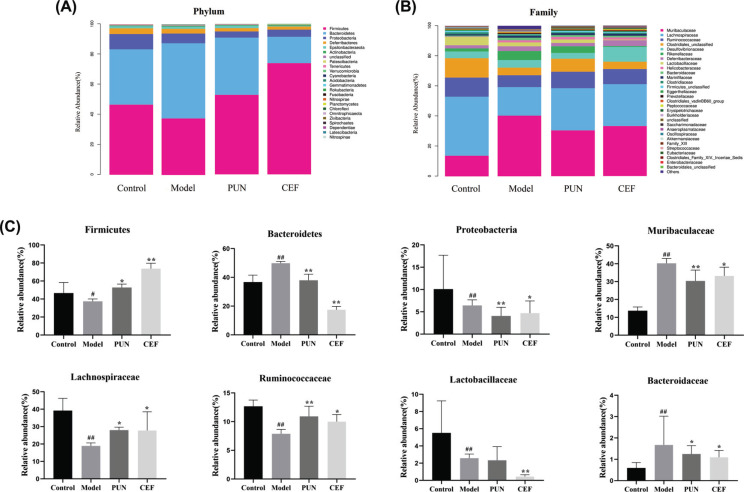
The composition of intestinal microbiota in each group of mice. (**A** and **B**) The abundance of phylum and family. (**C**) The bacteria differed significantly. Statistical significance: ^#^*p* < 0.05 and ^##^*p* < 0.01 compared with control group; *p < 0.05 and **p < 0.01 compared with model group (n = 6).

At the levels of phylum (Fig. **11A**) and department (Fig. **11B**), the response of the intestinal microbial composition to diarrhea and PUN treatment changed significantly. Fig. (**11C**) shows that, when compared to the model group, PUN treatment increased the relative abundance of Firmicutes while decreasing the relative abundance of Bacteroidetes (*p* <0.01). At the family level, the relative abundance of Muribaculaceae and Bacteroidaceae in the model group was higher than that in the other three groups. Simultaneously, the relative abundance of Lachnospiraceae, Ruminococcaceae, and Lactobacillaceae decreased in the model group compared to the control group but recovered after PUN treatment (*p* <0.05).

To further examine the changes in the functional spectrum of intestinal bacteria in response to the changes in the intestinal community, we used the PICRUSt software to analyze the composition of the KEGG pathway in the bacterial population. The relative abundance of the genes associated with nutrient metabolism in the model group increased significantly, which was the result compared to the control group (Table **4**). The genes included energy metabolism, metabolism of terpenoids, polyketides, amino acids, cofactors, vitamins, and nucleosides, biodegradation, and metabolism of xenobiotics, and biosynthesis and metabolism of glycan; medication restored them to the normal control level (*p* < 0.05). On the contrary, after drug treatment, carbohydrate metabolism and biosynthesis of other secondary metals in the model group decreased significantly, while the abundance of the genes increased significantly compared to their values in the control group (*p* < 0.05). However, lipid metabolism, transport, and catabolism did not change significantly before and after treatment. For proliferation-related genes, the gene abundance in signal transmission increased significantly after PUN treatment relative to that in the other groups (*p* < 0.01). The enteritis treatment group (Model, PUN, and CEF) had a significantly higher abundance of cellular processes and signaling, cell mobility, transcription, folding, sorting, degradation, metabolic diseases, cell growth, and cell death relative to the values of these parameters in the control group (*p* < 0.05). The abundance of replication and repair genes in the model group was higher than those in the other three groups (*p* < 0.01).

## 4 DISCUSSION

Acute bacterial diarrhea is a common infectious disease of the digestive tract. *Escherichia coli*, *Proteus*, and *Staphylococcus aureus* are the common pathogens of this disease; most of them are gram-negative bacilli [19]. When gram-negative bacilli enter the body, their endotoxin activates the inflammatory signal transduction pathway, causing the body to produce and release TNF-α, IL-6, and other inflammatory factors. [20]. Cefixime is a broad-spectrum cephalosporin antibiotic commonly used for treatment, it has a wide range of antibacterial effects and can inhibit DNA synthesis and replication, thus, causing sterilization. It is the preferred antibiotic for the treatment of bacterial enteritis. However, cefixime is not suitable for long-term application and interferes with the colonization resistance of gastrointestinal flora. It causes a severe imbalance in the bacterial flora and induces other complications [21]. Therefore, studying the mechanism of the development of bacterial enteritis and finding effective anti-enteritis drugs is very important.

In this study, five core genes of Pun, targeted toward bacterial enteritis, were screened, which included TNF, IL6, TP53, CASP3, and VEGFA, and the results were obtained by network pharmacology. The molecular docking of the ligands and proteins can intuitively present the structural morphology and chemical state of the drug-binding target. There are strong hydrogen bond interactions, which can promote the binding of small molecules to protein sites in amino acid active groups of Pun with TNF, IL6, TP53, CASP3, and VEGFA target proteins, and the results were obtained by molecular docking. The findings lend strong support to network pharmacology screening. Pun is thus a promising treatment compound.

The core targets of the PPI included TNF, IL6, TP53, CASP3, and VEGFA for the treatment of bacterial enteritis and were the key components of Pun. Activated T lymphocytes and macrophages mainly produce TNF. TNF can cause fever by directly stimulating the hypothalamic regulatory center to release IL-1. It can also stimulate other cells to produce IL-6 through IL-1 [22]. IL6 belongs to the interleukin family. It performs numerous functions, the most important of which is the regulation of responses associated with immunity and stress. It can bind to its receptor and cause a cascade of inflammatory reactions [23]. Previous studies showed that Pun could significantly reduce the production of IL-6, IL-8, and TNF-α in the gut, which were induced by LPS [24]. Pun inhibits the upstream medium NF-kB by inhibiting the production of IKBα and the phosphorylation of p65, according to molecular studies. NF-kB is linked to a traditional inflammatory pathway. When activated, this pathway can result in a large release of the downstream factors TNF and IL-6 [25]. Therefore, the mechanism of Pun in the treatment of bacterial enteritis is most likely related to the inflammatory pathway. TP53 is related to genes that affect apoptosis or regulate the cell cycle, such as the P21 gene, which encodes the cell cycle-dependent protein kinase inhibitor protein, and the BAX gene, which is a precursor protein that encodes apoptosis [26]. VEGFA is a member of the PDGF/VEGF growth factor family and has many functions, such as promoting cell migration, inhibiting apoptosis, and inducing vascular permeability [27]. CASP3 is a protein cleavage enzyme, called “death protease”, and is the key to apoptosis. Once activated, it can produce downstream cascade reactions, resulting in apoptosis of irreversibility of cells. IL-6 can activate CASP8 in the death receptor-mediated apoptosis pathway, further mediating CASP3 activation and leading to apoptosis [28, 29]. The increase in CASP3 is closely related to the occurrence of the intestinal inflammatory response, which is associated with the transduction pathway of inflammatory signaling, as found in previous studies [30]. IL-6 is closely related to inflammatory responses, intestinal inflammatory responses, and ulcerative colitis [31].

We analyzed the GO and KEGG pathways of Pun in the treatment of bacterial enteritis. Pun influenced multiple targets, structures, and forms, primarily related to cell proliferation, functional regulation, transcription factor activity, and so on, causing inflammatory and immune reactions during the occurrence and progression of bacterial enteritis. The results of the GO analysis confirmed the network pharmacology's logic and dependability. Pun primarily uses the PI3K-Akt and TNF signaling pathways, as well as other cancer and inflammation-related pathways identified through KEGG pathway analysis, to treat bacterial enteritis. The PI3K-Akt signaling pathway, is a signaling pathway for apoptosis, and its main functions include participation in cell differentiation, proliferation, apoptosis, and angiogenesis [32, 33]. Among the core targets of drug diseases, VEGFA is the key factor in the above two pathways. IL-6 is an important exogenous cytokine in the PI3K pathway. It can also activate the cell aging signal pathway and the TNF inflammatory response pathway, resulting in a series of reactions [34]. Additionally, PI3K can regulate the secretion of IL-6 by interacting with CD80/CD86 on the surface of dendritic cells [35].

The disturbance of normal intestinal microflora is one of the pathogenesis of bacterial enteritis [36]. In order to verify the anti-enteritis effect of PUN through core targets, a model of acute bacterial enteritis was developed by intragastric administration of pathogenic *Escherichia coli* O_101_. The body weight, fecal characteristics, and blood in the stool of the mice were recorded to calculate the disease activity index (DAI). The results showed that DAI increased significantly in the model group, indicating that the enteritis model was successfully established. Then, mice with enteritis were given a certain dose of PUN and CEF orally. After drug treatment, the DAI of mice was significantly decreased, indicating that angarin could improve enteritis induced by pathogenic bacteria. Pathological sections of the intestinal wall revealed granulocyte infiltration in the central axis of the intestinal villi and the lamina surrounding the intestinal glands, as well as a disorganized arrangement of the intestinal villi and an obvious inflammatory response. The importance of intestinal epithelial cells and tight junctions between cells (TJs) in the intestinal mucosal barrier is well understood. TJs comprise intestinal wall barrier proteins (ZO-1 and Occludin), which can regulate and maintain cell polarity and play a role in cell connectivity, and are directly related to intestinal barrier permeability. Pathogens can enter the body through paracellular junctions by altering barrier permeability and intestinal integrity by disrupting ZO-1 or Occludin structures [37, 38]. According to the histochemical results, ZO-1 and Occludin are expressed more in the control group's intestinal epithelium and significantly less in the model group compared to the control group. In the treated group, intestinal wall protein expression was significantly higher than in the model group. PUN was found to significantly improve intestinal wall permeability in mice with enteritis. When the intestinal epithelial barrier is compromised, intestinal flora migrates uncontrollably into the laminae propria, where surface antigens released by pathogens are recognized by TLR and NOD2 receptors on intestinal wall antigen-presenting cells, activating pro-inflammatory factors such as IKB and initiating inflammatory pathways such as NF-KB. Intestinal-associated lymphoid tissue (GALT) can be activated to promote the inflammatory response of local tissues by increasing the expression of IL-1β, IL-6, and TNF-α. Meanwhile, pro-inflammatory factors like IL-1β, IL-6, and TNF-α work with opportunistic pathogens to damage intestinal wall barrier proteins (ZO-1 and Occludin), causing tight junctions between cells to loosen and increasing intestinal barrier permeability. Through the damaged intestinal epithelium, LPS enters the intestinal wall and blood, inducing up-regulation of genes related to inflammatory factors in the intestinal wall, leading to an increase in inflammatory factors such as LPS, IL-1β, IL-6, and TNF-α in the blood, affecting the entire organism system [39, 40]. This experiment used ELISA, PCR, and WB to detect the expressions of PUN and the core targets of bacterial enteritis, *i.e*., TNF and IL-6, through network pharmacology. The results showed that PUN and CEF significantly down-regulated the expressions of TNF-α and IL-6 proteins in the serum and intestinal wall of mice with enteritis, alleviating the destruction of intestinal wall barrier proteins by inflammatory factors. Therefore, we inferred that PUN played a therapeutic role in bacterial enteritis by regulating related inflammatory signaling pathways.

Based on the above-mentioned results, Pun alleviated various symptoms of enteritis. In the normal human body, intestinal microbiota coexists and interacts to form a stable micro-ecosystem. Destroying the intestinal microbial population not only affects the digestion and absorption of food but also hinders the intestinal immune function, causing strong inflammatory reactions and the formation of lesions in the intestinal wall [41]. The changes in the intestinal microbiota were determined by the 16S rRNA analysis. The high-quality data generated indicated that it included all species in the community [42]. In terms of microbial loading, OTU analysis showed the microbial load of the control group mice was higher than that of the model group mice, and the treatment of Pun was able to restore the microbial load to normal levels. The model group mice had a lower α-diversity compared to the control group mice, which led to an imbalance in the intestinal flora of the model group mice. In this experiment, the α-diversity of the mice suffering from diarrhea could be restored to the level of the control group mice after seven days of Pun treatment. The α-diversity measures the diversity of microbiota in a single sample, whereas the β-diversity measures differences in species complexity at the population level. Bray Curtis distance is commonly used as a β-diversity index to determine structural changes in each group of gut microbiota [43, 44]. In terms of intestinal microbiota, we found significant differences between the model mice and the control mice. The results of the α-diversity analysis were consistent. The intestinal microbiota of the Pun-treated mice was similar to that of the control mice. Furthermore, PUN treatment homogenizes the intestinal microbiota probably due to the antibacterial properties of PUN, which has a broad spectrum and selectively inhibits harmful flora [45]. Therefore, PUN reshaped the intestinal microbiota of mice suffering from diarrhea and made it similar to that of the control mice. Based on flora composition at the phylum and family levels, we found that Pun inhibited the proliferation of Bacteroidetes, Muribaculaceae, Bacteroidaceae and enriched Firmicutes, Lachnospiraceae, Ruminococcaceae, and *Lactobacillus*. This is the mechanism by which PUN regulates the intestinal microbiota. Firmicutes, Lachnospiraceae, Ruminococcaceae, and *Lactobacillus* are chemotrophic bacteria that are conducive to the digestion and absorption of nutrients [46]. A decrease in the abundance of these bacteria often leads to dyspepsia. Bacteroidetes, Muribaculaceae, and Bacteroidaceae are conditional pathogens and are strongly associated with the occurrence of inflammatory bowel disease [47]. When the abundance of pathogenic bacteria increases, so do the levels of bacterial toxins and other harmful substances, which can aggravate the damage to the intestinal mucosal barrier and increase the permeability of the intestinal barrier. In animal experiments, the histochemical results of the tight junction proteins occludin and ZO-1 confirmed this. Cytotoxins and other harmful substances easily enter the body by crossing the barrier, stimulating immune cells to secrete many inflammatory mediators such as TNF-α, IL-6, *etc*., which upholds the previous findings of network pharmacology. These results suggested that changes in the intestinal microbiota may lead to changes in the functional spectrum of the entire intestinal microbiota. Thus, the regulation of the intestinal microbiota may be one of the mechanisms by which PUN exerts an anti-diarrhea effect [48]. Therefore, we used the PICRUSt software to predict the functional spectrum based on the taxa assigned by the OTU. We found that the model mice had more genes related to nutritional metabolism and cell proliferation than the control mice, and the Pun treatment returned them to the level of the control mice. This is because most pathogenic bacteria, which are anaerobic, and whose growth condition reflects the health of the intestinal flora, commonly show an imbalance. Once the intestinal epithelial barrier is damaged, oxygen enters the intestinal lumen, providing anaerobic bacteria a proliferative advantage. This eventually causes an imbalance in the intestinal microbiota. Excessive proliferation reduces the α-diversity, which is characterized by an imbalance in the intestinal microbiota [49, 50]. The model mice had more genes related to intestinal proliferation than the control mice. Pun treatment significantly increased the abundance of genes associated with signal transduction in mice from the control and enteritis groups, implying that Pun promoted interactions within the intestinal flora. These findings suggested that the two changes were associated with the functional spectrum and intestinal flora. Due to the excessive proliferation of microorganisms, the limited nutrients in the body are consumed, and most other benign microorganisms are inhibited, resulting in a decline in the diversity of microbial species in mice with enteritis [51]. The genes related to nutritional metabolism were significantly lower in the mice with enteritis, while Pun treatment increased the abundance of these genes.

## CONCLUSION

To sum up, this study screened important targets through network pharmacological analysis, which are the targets of Pun in the treatment of bacterial enteritis. Furthermore, the study determined the efficacy of Pun based on the results of molecular docking and animal experiments, as well as by sequencing the intestinal flora to investigate the effect of Pun on the intestinal flora of mice suffering from bacterial enteritis. Preliminary results showed that the main active component of Pun might improve enteritis and the imbalance in the intestinal flora by influencing multiple targets, multiple structures, and multiple forms, reducing the inflammatory reaction in the mouse intestinal wall, and protecting the intestinal wall that acts as a barrier. Pun was found to be an effective antibacterial and anti-inflammatory agent in this study. The results might provide a theoretical basis to further investigate and elucidate the mechanism of the components of Pun for more efficient treatment of bacterial enteritis. The research also served as a resource for the development, application, and comprehensive understanding of traditional Chinese medicines.

## AUTHORS’ CONTRIBUTIONS

S.H., Q.Z. performed the experiments, analyzed the data, and wrote the manuscript; Y.W. and H.G. performed the experiments; Z.H. and S.Z. contributed to the study design and overall supervision. All authors reviewed the manuscript. S.H. and Y.W. contributed equally to this work.

## Figures and Tables

**Fig. (4):**
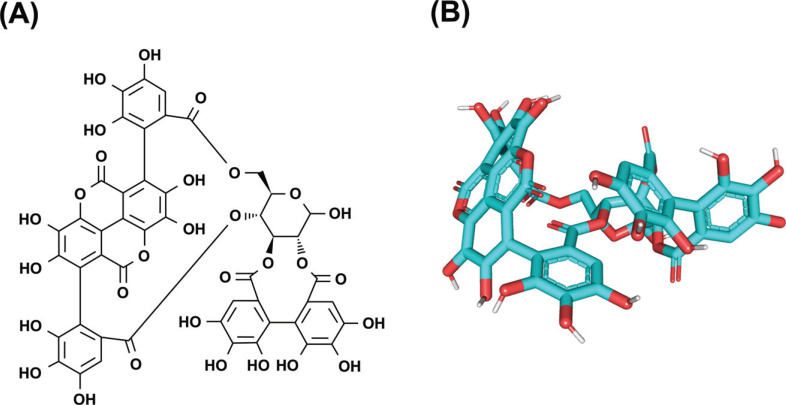
The structure of Pun. (**A**) 2D diagram and (**B**) 3D diagram.

**Table 1:** Website of database and application software.

**Name of Database and Software**	**Web Address**
TCMSP database	https://old.tcmsp-e.com/tcmsp.php/
UniProt database	https://www.uniprot.org/
GeneCards database	https://www.genecards.org/
STRING database	https://string-db.org/
DAVID database	https://david.ncifcrf.gov/
PubChem	https://pubchem.ncbi.nlm.nih.gov/
RCSB PDB	https://www.rcsb.org/
Venny	https://bioinfogp.cnb.csic.es/tools/venny/
Cytoscape 3.9.0 software	http://www.cytoscape.org/
AutoDock Vina	https://vina.scripps.edu/
PyMOL 2.2.0 software	https://pymol.org/2/
PICRUSt software	https://picrust.github.io/picrust/

**Table 2:** Potential active components of PUN.

**Mol ID**	**Molecule Name**	**MW**	**OB (%)**	**DL**
MOL001002	Ellagic acid	302.2	43.06	0.43
MOL000358	β-sitosterol	414.79	36.91	0.75
MOL000422	Kaempferol	286.25	41.88	0.24
MOL000492	(+)-catechin	290.29	54.83	0.24
MOL000006	Luteolin	286.25	36.16	0.25
MOL000098	Quercetin	302.25	46.43	0.28

**Table 3:** The results of molecular docking.

**Target**	**PDB**	**Energy (kcal/mol)**	**Hydrogen Bond Number**
TNF	6CGI	-10.9	5
IL6	5FUC	-9.3	10
TP53	5AOM	-9.2	9
CASP3	1NMS	-10.2	9
VEGFA	5DN2	-10.3	11

**Table 4:** KEGG pathways of functional spectrum of intestinal bacteria.

**KEGG Pathways**	**Relative Abundance (%)**			
	**Control**	**Model**	**PUN**	**CEF**
Carbohydrate Metabolism	11.32 ± 1.34	4.65 ± 2.32^##^	8.33 ± 1.90**	8.32 ± 1.47**
Energy Metabolism	2.49 ± 1.21	5.47 ± 0.64^#^	4.29 ± 0.74*	4.40 ± 0.60*
Metabolism of Terpenoids and Polyketides	0.65 ± 0.32	1.47 ± 0.15^##^	1.13 ± 0.22*	1.08 ± 0.19*
Metabolism	0.95 ± 0.47	2.18 ± 0.25^#^	1.65 ± 0.31**	1.69 ± 0.26**
Metabolism of Cofactors and Vitamins	1.76 ± 0.85	3.81 ± 0.49^#^	3.02 ± 0.56**	3.07 ± 0.39**
Cellular Processes and Signaling	1.92 ± 0.99	4.68 ± 0.66^##^	3.49 ± 0.68**	3.51 ± 0.72**
Cell Motility	1.79 ± 1.15	4.72 ± 0.87^##^	3.11 ± 0.77*	3.31 ± 0.91*
Metabolism of Other Amino Acids	0.67 ± 0.32	1.45 ± 0.12^##^	1.14 ± 0.18*	1.13 ± 0.17*
Nucleotide Metabolism	2.01 ± 1.02	4.73 ± 0.49^#^	3.54 ± 0.62*	3.49 ± 0.68*
Lipid Metabolism	2.92 ± 0.32	2.23 ± 0.40	1.28 ± 0.62	2.20 ± 0.42
Signal Transduction	1.00 ± 0.57	1.73 ± 0.34^#^	2.51 ± 0.34**	1.88 ± 0.37*
Amino Acid Metabolism	4.43 ± 2.24	9.92 ± 1.25^##^	7.71 ± 1.45**	7.64 ± 1.25**
Transcription	1.38 ± 0.75	3.76 ± 0.57^##^	2.62 ± 0.52*	2.58 ± 0.76*
Transport and Catabolism	0.14 ± 0.06	0.20 ± 0.04	0.22 ± 0.03	0.20 ± 0.02
Environmental Adaptation	0.24 ± 0.04	0.10 ± 0.06	0.17 ± 0.04*	0.18 ± 0.04*
Folding, Sorting, and Degradation	1.28 ± 0.63	2.74 ± 0.24^#^	2.17 ± 0.39*	2.13 ± 0.27*
Biosynthesis of Other Secondary Metabolites	1.00 ± 0.17	0.46 ± 0.23^#^	0.79 ± 0.16*	0.79 ± 0.17*
Metabolic Diseases	0.05 ± 0.03	0.11 ± 0.01^##^	0.09 ± 0.02**	0.08 ± 0.01**
Cell Growth and Death	0.29 ± 0.14	0.63 ± 0.07^#^	0.48 ± 0.09*	0.48 ± 0.08*
Membrane Transport	15.28 ± 2.62	5.31 ± 2.84^##^	10.10 ± 1.87**	10.68 ± 3.25**
Xenobiotics Biodegradation and Metabolism	0.49 ± 0.20	1.09 ± 0.08^##^	0.84 ± 0.08**	0.87 ± 0.06**
Glycan Biosynthesis and Metabolism	1.13 ± 0.54	2.08 ± 0.18^##^	1.77 ± 0.33*	1.72 ± 0.20*

**Note: **^#^*p* < 0.05 and ^##^*p* < 0.01 compared with control group; *p < 0.05 and **p < 0.01 compared with model group.

## Data Availability

The data used to support the findings of this study are included in the article.

## LIST OF ABBREVIATIONS

BP: Biological Process
CASP-3: Caspase-3
CC: Cellular Component
CEF: Cefixime
DAI: Disease Activity Index
DL: Drug Likeness
ELISA: Enzyme Linked Immunosorbent Assay
Go: Gene Ontology
HE: Hematoxylin and Eosin
IL-6: Interleukin-6
KEGG: Kyoto Encyclopedia of Genes and Genomes
MF: Molecular Function
OB: Oral Bioavailability
PPI: Protein Protein Interaction
Pun: Punicalagin
TCMSP: Traditional Chinese Medicine Systems Pharmacology
TJs: Tight Junctions
TNF-α: Tumor Necrosis Factor-α
TP53: Tumor Suppressor p53
VEGFA: Vascular Endothelial Growth Factor A
WB: Western Blot
ZO-1: Zonula Occludens-1

## References

[1] Ashkenazi S., Schwartz E. (2020). Traveler’s diarrhea in children: New insights and existing gaps.. Travel Med. Infect. Dis..

[2] Bi C., Jing W., Xie X., Liu Y. (2021). Efficacy and mechanism of traditional Chinese medicine in relieving antibiotic-resistant bacterial diarrhea in children: Study protocol for a randomized controlled trial.. Trials.

[3] Westermarck E. (2016). Chronic diarrhea in dogs: What do we actually know about it?. Top. Companion Anim. Med..

[4] Li G., Feng Y., Xu Y., Wu Q., Han Q., Liang X., Yang B., Wang X., Xia X. (2015). The anti-infective activity of punicalagin against Salmonella enterica subsp. enterica serovar typhimurium in mice.. Food Funct..

[5] Bialonska D., Kasimsetty S.G., Schrader K.K., Ferreira D. (2009). The effect of pomegranate (Punica granatum L.) byproducts and ellagitannins on the growth of human gut bacteria.. J. Agric. Food Chem..

[6] Brighenti V., Iseppi R., Pinzi L., Mincuzzi A., Ippolito A., Messi P., Sanzani S.M., Rastelli G., Pellati F. (2021). Antifungal activity and DNA topoisomerase inhibition of hydrolysable tannins from Punica granatum L.. Int. J. Mol. Sci..

[7] Neyrinck A.M., Van Hée V.F., Bindels L.B., De Backer F., Cani P.D., Delzenne N.M. (2013). Polyphenol-rich extract of pomegranate peel alleviates tissue inflammation and hypercholesterolaemia in high-fat diet-induced obese mice: potential implication of the gut microbiota.. Br. J. Nutr..

[8] Akdis C.A. (2021). Does the epithelial barrier hypothesis explain the increase in allergy, autoimmunity and other chronic conditions?. Nat. Rev. Immunol..

[9] Ru J., Li P., Wang J., Zhou W., Li B., Huang C., Li P., Guo Z., Tao W., Yang Y., Xu X., Li Y., Wang Y., Yang L. (2014). TCMSP: A data-base of systems pharmacology for drug discovery from herbal medicines.. J. Cheminform..

[10] Guo W., Huang J., Wang N., Tan H.Y., Cheung F., Chen F., Feng Y. (2019). Integrating network pharmacology and pharmacological evaluation for deciphering the action mechanism of herbal formula zuojin pill in suppressing hepatocellular carcinoma.. Front. Pharmacol..

[11] Szklarczyk D., Gable A.L., Nastou K.C., Lyon D., Kirsch R., Pyysalo S., Doncheva N.T., Legeay M., Fang T., Bork P., Jensen L.J., von Mering C. (2021). The STRING database in 2021: customizable protein–protein networks, and functional characterization of user-uploaded gene/measurement sets.. Nucleic Acids Res..

[12] The Gene Ontology Consortium (2017). Expansion of the Gene Ontology knowledgebase and resources.. Nucleic Acids Res..

[13] Kanehisa M., Furumichi M., Tanabe M., Sato Y., Morishima K. (2017). KEGG: New perspectives on genomes, pathways, diseases and drugs.. Nucleic Acids Res..

[14] Aucar M.G., Cavasotto C.N. (2020). Molecular docking using quantum mechanical-based methods.. Methods Mol. Biol..

[15] Xu B., Yan Y., Huang J., Yin B., Pan Y., Ma L. (2020). Cortex Phellodendri extract's anti-diarrhea effect in mice related to its modification of gut microbiota.. Biomedecine & pharmacotherapie.

[16] Chen R., Wang J., Zhan R., Zhang L., Wang X. (2019). Fecal metabonomics combined with 16S rRNA gene sequencing to analyze the changes of gut microbiota in rats with kidney-yang deficiency syndrome and the intervention effect of You-gui pill.. J. Ethnopharmacol..

[17] Hsin K.Y., Ghosh S., Kitano H. (2013). Combining machine learning systems and multiple docking simulation packages to improve docking prediction reliability for network pharmacology.. PLoS One.

[18] Wu H., Chen Q. Y., Wang W. Z., Chu S., Liu X. X., Liu Y. J., Tan C., Zhu F., Deng S. J., Dong Y. L., Yu T., Gao F., He H. X., Leng X. Y., Fan H. (2021). Compound sophorae decoction enhances intestinal barrier function of dextran sodium sulfate induced colitis via regulating notch signaling pathway in mice.. Biomedecine & pharmacotherapie.

[19] Bandsma R.H.J., Sadiq K., Bhutta Z.A. (2019). Persistent diarrhoea: Current knowledge and novel concepts.. Paediatr. Int. Child Health.

[20] Arasaradnam R.P., Brown S., Forbes A., Fox M.R., Hungin P., Kelman L., Major G., O’Connor M., Sanders D.S., Sinha R., Smith S.C., Thomas P., Walters J.R.F. (2018). Guidelines for the investigation of chronic diarrhoea in adults: British Society of Gastroenterology, 3rd edition.. Gut.

[21] Szajewska H., Kołodziej M. (2015). Systematic review with meta-analysis: Saccharomyces boulardii in the prevention of antibiotic-associated diarrhoea.. Aliment. Pharmacol. Ther..

[22] Chen T., Zhang X., Zhu G., Liu H., Chen J., Wang Y., He X. (2020). Quercetin inhibits TNF-α induced HUVECs apoptosis and inflammation via downregulating NF-kB and AP-1 signaling pathway in vitro.. Medicine.

[23] Jing B., Wang T., Sun B., Xu J., Xu D., Liao Y., Song H., Guo W., Li K., Hu M., Zhang S., Ling J., Kuang Y., Zhang T., Zhou B.P., Yao F., Deng J. (2020). IL6/STAT3 signaling orchestrates premetastatic niche formation and immunosuppressive traits in lung.. Cancer Res..

[24] Cao Y., Chen J., Ren G., Zhang Y., Tan X., Yang L. (2019). Punicalagin prevents inflammation in LPS-induced RAW264.7 macrophages by inhibiting FoxO3a/Autophagy signaling pathway.. Nutrients.

[25] Peng L., Wen L., Shi Q.F., Gao F., Huang B., Meng J., Hu C.P., Wang C.M. (2020). Scutellarin ameliorates pulmonary fibrosis through inhibiting NF-κB/NLRP3-mediated epithelial–mesenchymal transition and inflammation.. Cell Death Dis..

[26] Donehower L.A., Soussi T., Korkut A., Liu Y., Schultz A., Cardenas M., Li X., Babur O., Hsu T.K., Lichtarge O., Weinstein J.N., Akbani R., Wheeler D.A. (2019). Integrated analysis of TP53 gene and pathway alterations in the cancer genome atlas.. Cell Rep..

[27] Vila Ellis L., Cain M.P., Hutchison V., Flodby P., Crandall E.D., Borok Z., Zhou B., Ostrin E.J., Wythe J.D., Chen J. (2020). Epithelial vegfa specifies a distinct endothelial population in the mouse lung.. Dev. Cell.

[28] Hu L., Chen M., Chen X., Zhao C., Fang Z., Wang H., Dai H. (2020). Chemotherapy-induced pyroptosis is mediated by BAK/BAX-caspase-3-GSDME pathway and inhibited by 2-bromopalmitate.. Cell Death Dis..

[29] Kuo W.T., Shen L., Zuo L., Shashikanth N., Ong M.L.D.M., Wu L., Zha J., Edelblum K.L., Wang Y., Wang Y., Nilsen S.P., Turner J.R. (2019). Inflammation-induced occludin downregulation limits epithelial apoptosis by suppressing Caspase-3 expression.. Gastroenterology.

[30] Sheahan B.J., Freeman A.N., Keeley T.M., Samuelson L.C., Roper J., Hasapis S., Lee C.L., Dekaney C.M. (2021). Epithelial regeneration after doxorubicin arises primarily from early progeny of active intestinal stem cells.. Cell. Mol. Gastroenterol. Hepatol..

[31] Siersbæk R., Scabia V., Nagarajan S., Chernukhin I., Papachristou E.K., Broome R., Johnston S.J., Joosten S.E.P., Green A.R., Kumar S., Jones J., Omarjee S., Alvarez-Fernandez R., Glont S., Aitken S.J., Kishore K., Cheeseman D., Rakha E.A., D’Santos C., Zwart W., Russell A., Brisken C., Carroll J.S. (2020). IL6/STAT3 signaling hijacks estrogen receptor α enhancers to drive breast cancer metastasis.. Cancer Cell.

[32] Jia X., Wen Z., Sun Q., Zhao X., Yang H., Shi X., Xin T. (2019). Apatinib suppresses the proliferation and apoptosis of gastric cancer cells via the PI3K/Akt signaling pathway.. J. BUON.

[33] Chen Y.H., Yang S.F., Yang C.K., Tsai H.D., Chen T.H., Chou M.C., Hsiao Y.H. (2020). Metformin induces apoptosis and inhibits migration by activating the AMPK/p53 axis and suppressing PI3K/AKT signaling in human cervical cancer cells.. Mol. Med. Rep..

[34] Li S., Dai Q., Zhang S., Liu Y., Yu Q., Tan F., Lu S., Wang Q., Chen J., Huang H., Liu P., Li M. (2018). Ulinastatin attenuates LPS-induced inflammation in mouse macrophage RAW264.7 cells by inhibiting the JNK/NF-κB signaling pathway and activating the PI3K/Akt/Nrf2 pathway.. Acta Pharmacol. Sin..

[35] Zhang X., Hu F., Li G., Li G., Yang X., Liu L., Zhang R., Zhang B., Feng Y. (2018). Human colorectal cancer-derived mesenchymal stem cells promote colorectal cancer progression through IL-6/JAK2/STAT3 signaling.. Cell Death Dis..

[36] Tuganbaev T., Mor U., Bashiardes S., Liwinski T., Nobs S.P., Leshem A., Dori-Bachash M., Thaiss C.A., Pinker E.Y., Ratiner K., Adlung L., Federici S., Kleimeyer C., Moresi C., Yamada T., Cohen Y., Zhang X., Massalha H., Massasa E., Kuperman Y., Koni P.A., Harmelin A., Gao N., Itzkovitz S., Honda K., Shapiro H., Elinav E. (2020). Diet diurnally regulates small intestinal microbiome-epithelial-immune homeostasis and enteritis.. Cell.

[37] Zihni C., Mills C., Matter K., Balda M.S. (2016). Tight junctions: from simple barriers to multifunctional molecular gates.. Nat. Rev. Mol. Cell Biol..

[38] Kim S., Kim G.H. (2017). Roles of claudin-2, ZO-1 and occludin in leaky HK-2 cells.. PLoS One.

[39] Gomes A.C., Hoffmann C., Mota J.F. (2018). The human gut microbiota: Metabolism and perspective in obesity.. Gut Microbes.

[40] Camilleri M., Madsen K., Spiller R., Van Meerveld B.G., Verne G.N. (2012). Intestinal barrier function in health and gastrointestinal disease.. Neurogastroenterol. Motil..

[41] Wisniewski P.J., Dowden R.A., Campbell S.C. (2019). Role of dietary lipids in modulating inflammation through the gut microbiota.. Nutrients.

[42] Laudadio I., Fulci V., Palone F., Stronati L., Cucchiara S., Carissimi C. (2018). Quantitative assessment of shotgun metagenomics and 16S rDNA amplicon sequencing in the study of human gut microbiome.. OMICS.

[43] Pei Z., Xiaowei W., Yajuan L., Yuanhong X. (2021). Exploring the characteristics of intestinal microbiota in hematologic malignancy patients via 16s rDNA high-throughput sequencing.. Clin. Lab..

[44] Dong S., jiao J., Jia S., Li G., Zhang W., Yang K., Wang Z., Liu C., Li D., Wang X. (2021). 16S rDNA full-length assembly sequencing technology analysis of intestinal microbiome in polycystic ovary syndrome.. Front. Cell. Infect. Microbiol..

[45] Mun S.H., Kang O.H., Kong R., Zhou T., Kim S.A., Shin D.W., Kwon D.Y. (2018). Punicalagin suppresses methicillin resistance of Staphylococcus aureus to oxacillin.. J. Pharmacol. Sci..

[46] Megrian D., Taib N., Witwinowski J., Beloin C., Gribaldo S. (2020). One or two membranes? Diderm Firmicutes challenge the Gram‐positive/Gram‐negative divide.. Mol. Microbiol..

[47] Larsbrink J., McKee L.S. (2020). Bacteroidetes bacteria in the soil: Glycan acquisition, enzyme secretion, and gliding motility.. Adv. Appl. Microbiol..

[48] Yan X., Jin J., Su X., Yin X., Gao J., Wang X., Zhang S., Bu P., Wang M., Zhang Y., Wang Z., Zhang Q. (2020). Intestinal flora modulates blood pressure by regulating the synthesis of intestinal-derived corticosterone in high salt-induced hypertension.. Circ. Res..

[49] Milani C., Duranti S., Bottacini F., Casey E., Turroni F., Mahony J., Belzer C., Delgado Palacio S., Arboleya Montes S., Mancabelli L., Lugli G.A., Rodriguez J.M., Bode L., de Vos W., Gueimonde M., Margolles A., van Sinderen D., Ventura M. (2017). The first microbial colonizers of the human gut: Composition, activities, and health implications of the infant gut microbiota.. Microbiol. Mol. Biol. Rev..

[50] Shin N.R., Whon T.W., Bae J.W. (2015). Proteobacteria: Microbial signature of dysbiosis in gut microbiota.. Trends Biotechnol..

[51] Gresse R., Chaucheyras-Durand F., Fleury M.A., Van de Wiele T., Forano E., Blanquet-Diot S. (2017). Gut microbiota dysbiosis in postweaning piglets: Understanding the keys to health.. Trends Microbiol..

